# Depth of Sequencing Plays a Determining Role in the Characterization of Phage Display Peptide Libraries by NGS

**DOI:** 10.3390/ijms24065396

**Published:** 2023-03-11

**Authors:** Ane Beth Sloth, Babak Bakhshinejad, Camilla Stavnsbjerg, Maria Rossing, Andreas Kjaer

**Affiliations:** 1 Department of Clinical Physiology and Nuclear Medicine & Cluster for Molecular Imaging, Copenhagen University Hospital—Rigshospitalet & Department of Biomedical Sciences, University of Copenhagen, 2200 Copenhagen, Denmark; 2 Center for Genomic Medicine, Rigshospitalet, Copenhagen University Hospital, 2100 Copenhagen, Denmark; 3 Department of Clinical Medicine, Faculty of Health and Medical Sciences, University of Copenhagen, 2200 Copenhagen, Denmark

**Keywords:** composition, deep sequencing, distinguishing capacity, diversity, illumina sequencing, next-generation sequencing, phage display peptide library, Ph.D.^TM^-12 peptide library, quality, sequencing depth

## Abstract

Next-generation sequencing (NGS) has raised a growing interest in phage display research. Sequencing depth is a pivotal parameter for using NGS. In the current study, we made a side-by-side comparison of two NGS platforms with different sequencing depths, denoted as lower-throughput (LTP) and higher-throughput (HTP). The capacity of these platforms for characterization of the composition, quality, and diversity of the unselected Ph.D.^TM^-12 Phage Display Peptide Library was investigated. Our results indicated that HTP sequencing detects a considerably higher number of unique sequences compared to the LTP platform, thus covering a broader diversity of the library. We found a larger percentage of singletons, a smaller percentage of repeated sequences, and a greater percentage of distinct sequences in the LTP datasets. These parameters suggest a higher library quality, resulting in potentially misleading information when using LTP sequencing for such assessment. Our observations showed that HTP reveals a broader distribution of peptide frequencies, thus revealing increased heterogeneity of the library by the HTP approach and offering a comparatively higher capacity for distinguishing peptides from each other. Our analyses suggested that LTP and HTP datasets show discrepancies in their peptide composition and position-specific distribution of amino acids within the library. Taken together, these findings lead us to the conclusion that a higher sequencing depth can yield more in-depth insights into the composition of the library and provide a more complete picture of the quality and diversity of phage display peptide libraries.

## 1. Introduction

Phage display, introduced in 1985 by George Smith [1], has been used extensively in the identification of novel ligands with potential applications in different research areas including, but not limited to, drug discovery [2], drug delivery [3], and molecular imaging [4]. In recent decades, combinatorial phage display libraries have raised growing attention for the translation of peptide-based diagnostics and therapeutics into the clinic. These libraries are constructed by in-frame cloning of random oligonucleotides into the surface protein gene of a bacteriophage, leading to the expression of a vast number of exogenous peptides as fusions to the corresponding surface protein. This links the phenotype to the genotype, allowing for sequencing of the relevant part of the phage genome to retrieve the displayed peptide sequence [5].

The advent of phage display peptide libraries has enabled the high-throughput screening on a variety of important cellular and molecular targets [6,7,8,9,10]. Random peptide sequences expressed on the phage surface can interact with the target of interest and phages expressing peptides that bind specifically to the target can be isolated. Phage display screening, called biopanning, consists of iterative rounds in which the library is presented to the target of interest, and the selected phage pool is amplified by infection of *Escherichia coli* cells. The amplified phages serve as input for the subsequent round of biopanning [11]. Traditionally, biopanning outputs have been analyzed by Sanger sequencing. This sequencing approach can identify only a limited number of peptides in the library. Recent advances have combined phage display with next-generation sequencing (NGS), allowing for the sequencing of thousands to millions of peptide inserts in parallel [12,13,14]. This eliminates the laborious procedure of picking random clones and provides increased resolution of the biopanning output compared to Sanger sequencing. NGS allows researchers to analyze the composition of large peptide libraries on a deeper scale. This is valuable for monitoring, troubleshooting, and evaluating the design and construction of unselected naïve libraries, gaining detailed insights into the impact of the selective pressure applied to the library, and obtaining a solid understanding of the evolution of peptide populations during library selection [15]. NGS has not only been performed on biopanning eluates [16,17,18] but has also been exploited to investigate unselected and amplified phage display libraries [13,14,19,20,21]. A pivotal parameter for using NGS is the sequencing depth. Technically, depth (also referred to as depth of coverage) is defined as the number of times a given nucleotide has been read during the sequencing reaction [22]. In the context of peptide phage display, the depth of sequencing represents the number of reads that contain the displayed peptide-encoding sequence. Different factors influence the required depth of sequencing for the analysis of phage display peptide libraries. Nevertheless, the optimal sequencing depth is mainly dependent on the complexity of the peptide pool [13]. It is an accepted view that a higher sequencing depth provides a more representative coverage of the library and improves the accuracy by which the composition of the library can be characterized [23], thus leading to a more in-depth understanding of the library quality and the selection process.

In phage display experiments, the highest possible number of library members should be investigated. Hence, it is of particular importance to use a higher sequencing depth to characterize as much of the library diversity as possible as well as to evaluate the composition and quality of the library more accurately. Some statistical methods have been reported to measure the diversity of phage display peptide libraries [24]. These analyses have been conducted based on sampling a limited pool of the library by Sanger sequencing. Here, we used the number of unique sequences as an equivalent to diversity. The definition of diversity as the number of unique sequences is a commonly accepted idea in phage display literature [16,19,25,26,27,28,29,30], motivated by increasing number of NGS-based phage display works in recent years which allow for sampling of a larger sequence space. In the current study, we investigated the capacity of two sequencing depths (each on two different input volumes) of NGS to characterize the unselected Ph.D.^TM^-12 phage display peptide library. In a broad sense, low-throughput (LTP) sequencing refers to Sanger sequencing and high-throughput (HTP) sequencing refers to all the different types of NGS platforms. In this work, we denote LTP as the NGS platform with the lower throughput (MiSeq Reagent Kit v2) and HTP as the NGS platform with the higher throughput (NextSeq 500/550 Mid Output Kit v.2.5). The aim of the current study was to provide an evaluation of the impact of sequencing depth on the analysis of diversity, quality, and composition of an unselected phage display peptide library. Our findings indicated that a higher sequencing depth offers a more rigorous evaluation of the library diversity and quality, uncovers a more heterogeneous distribution of peptide frequencies, provides a comparatively higher capacity for the differentiation of peptide frequencies, and results in the detection of distinct amino acid and peptide compositions for the library compared to sequencing modality with the lower depth. Based on the results, we discuss how the sequencing depth can lead to different outcomes for the characteristics of the library, alter the direction of analyses, and make a major impact on the final conclusions drawn from phage display data.

## 2. Results

### 2.1. Higher Sequencing Depth Detects a Larger Diversity of the Library 

DNA isolated from two different volumes (1 µL and 10 µL) of the Ph.D.^TM^-12 library was sequenced using higher- and lower-throughput (HTP and LTP, respectively) sequencing platforms. According to NEB, 1 µL and 10 µL of the library represent 1 × 10^10^ and 1 × 10^11^ phage particles, respectively. Table 1 shows the results of the NGS analysis including the number and percentage of cleaned and removed reads for each sample. For samples sequenced by HTP, the number of unique cleaned reads is approximately 3.7 × 10^6^, whereas, for the samples sequenced by the LTP platform, the number of unique cleaned reads is 5.2 × 10^5^ and 6.7 × 10^5^ reads for 1 μL and 10 μL, respectively. The data show that HTP sequencing can cover a bigger diversity of the library pool. This can be observed for both volumes. Compared to the LTP platform, HTP sequencing provides a 7.1 times higher number of unique cleaned reads in 1 µL sample and a 5.5 times higher number of unique cleaned reads in 10 µL sample.

### 2.2. Higher Sequencing Depth Reveals a Smaller Percentage of Singletons in the Library 

**Figure 1:**
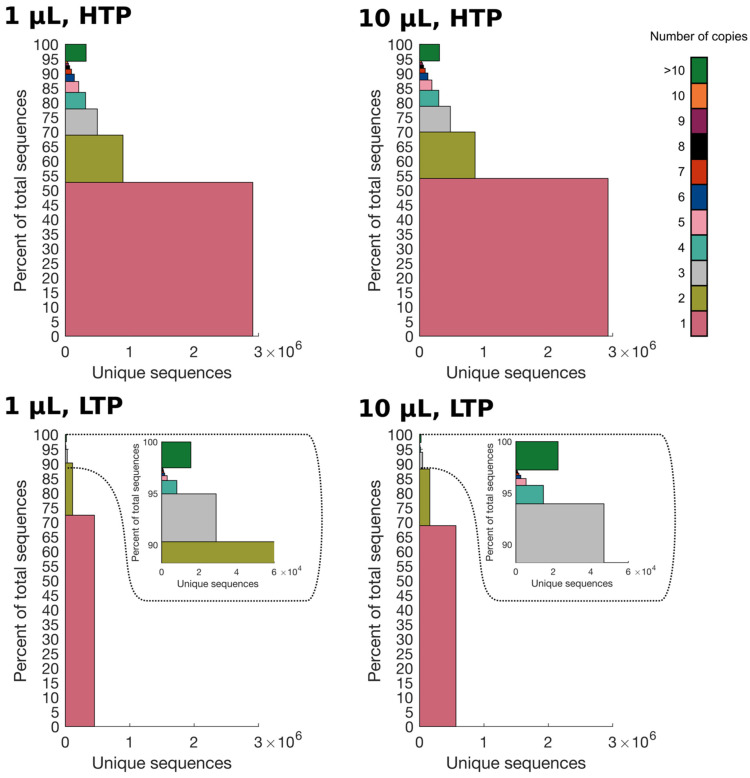
Stacked bar plot for HTP datasets (1 µL and 10 µL) and LTP datasets (1 µL and 10 µL). The datasets were grouped into color-coded populations according to the absolute frequency of peptide sequences (see color key). For each population, the sum of abundances is shown as the height for each segment, whereas the width corresponds to the number of unique sequences in each segment. Chi-square test with Yates correction proved the difference between the singleton populations to be statistically significant for all four samples (*p* < 0.0001).

Each sample was grouped into bins based on the absolute frequencies of peptide sequences (1, 2, 3, 4, 5, 6, 7, 8, 9, 10, and >10). Stacked bar plots were generated for each sample, showing the percentage of reads in the different categories. As seen from the horizontal axis in Figure 1, HTP sequencing can distinguish a broader diversity of the library. Furthermore, the vertical axis indicates that the singleton population makes up a smaller proportion of the library in HTP compared to LTP datasets. When comparing 1 μL HTP to 1 μL LTP, the singleton population makes up 52.7 % for HTP and 72.4 % for LTP (19.7 percentage points difference). That also holds true for comparison of 10 μL HTP and 10 μL LTP (54 % and 68.8 %, respectively, showing 14.8 percentage points difference). The differences between the singleton populations are statistically significant (*p* < 0.0001). The differences between the two HTP datasets (1.3 percentage points) and the two LTP datasets (3.6 percentage points) are also statistically significant (*p* < 0.0001), despite their smaller differences. This is caused by the large number of reads for each sample. Differences in the color-coded populations can be observed for the 11 bins. Once the bins of singleton and doubleton populations are combined, they sum to 88.2 % of the library in 10 µL LTP, and 69.9 % of the library in 10 µL HTP. By merging the bins of peptides with frequencies from 1 to 10, HTP sequencing still represents a lower proportion of the library compared to the LTP platform (94.3 % for 10 µL HTP and 97.3 % for 10 µL LTP, respectively). Taken together, a larger diversity of the library (larger width of blocks) and a smaller percentage of singletons (smaller height of the pink block) in the two HTP datasets suggest that a bigger heterogeneity can be observed for HTP compared to LTP datasets. 

The percentage of distinct sequences, representing the relative diversity normalized to each dataset, was calculated for each sample by dividing the number of unique cleaned reads by the number of absolute cleaned reads (Table 1). No substantial difference can be observed between the two HTP datasets (67.12 % for 1 µL and 68.12 % for 10 µL), and the two LTP datasets (83.52 % for 1 µL and 81.24 % for 10 µL). However, a higher percentage of distinct sequences can be observed once LTP datasets are compared to HTP datasets. 

### 2.3. Higher Sequencing Depth Uncovers a More Heterogeneous Distribution of Absolute Frequencies 

**Figure 2:**
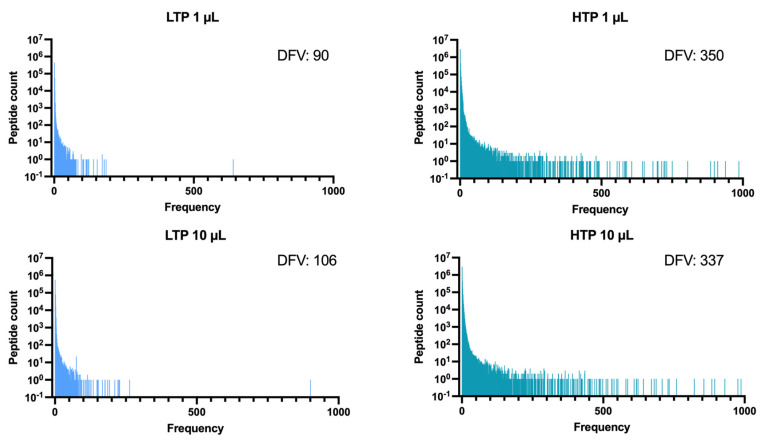
The distribution of absolute frequencies for each sample. For each dataset LTP (1 μL and 10 μL) and HTP (1 μL and 10 μL), the number of peptides with absolute frequencies from 1 to >1000 was identified. The number of distinct frequency values (DFVs) were counted for each sample.

To investigate the distribution of absolute frequencies for different sequencing depths, the absolute frequencies for each peptide were counted for both LTP and HTP datasets. As indicated in Figure 2, most peptides have low absolute frequencies (<100), and only a few peptides are found with absolute frequencies >500. This trend is observed for both LTP and HTP datasets, even though the absolute frequency values are generally lower for LTP compared to HTP datasets. A significant difference in the number of peptides is observed once LTP and HTP datasets are compared. Interestingly, HTP identifies a significantly higher count of peptides compared to LTP and the discrepancy between these datasets is more evident in higher absolute frequencies. Of note, even the 1 µL HTP dataset can reveal more peptides than the 10 µL LTP dataset. Only slight differences in the number of peptides per frequency were observed for different volumes (1 μL and 10 μL) of the same depth.

To obtain a better picture of the frequency distribution and evaluate the diversity of frequencies represented by different sequencing depths and the two investigated volumes, the number of distinct frequency values (DFVs) was determined for each sample as the count of frequency points for each dataset (the horizontal axis on Figure 2). As shown in Figure 2, the HTP datasets (1 µL and 10 µL) represent a higher number of DFVs compared to the LTP datasets (1 µL and 10 µL), which indicates the detection of a wider range and more heterogeneous distribution of frequencies in the higher depth of sequencing. 

### 2.4. Higher Sequencing Depth Can Provide a Comparatively Higher Distinguishing Capacity for the Detection of Peptide Frequencies 

An analysis was performed to compare the capacity of LTP and HTP platforms to distinguish peptide frequencies from each other. Peptides with absolute frequencies of 5, 10, 15, etc. up to 50 were noted in the 10 μL LTP dataset. Given the large number of peptides with absolute frequencies 5, 10, and 15 (1194 peptides in total), absolute frequencies 20, 25, 30, 35, 40, 45, and 50 were included in the analysis (51 peptides in total). Subsequently, the peptide sequences were retrieved in the 10 μL HTP dataset, and the absolute frequencies were noted. The 95% confidence intervals for the absolute frequencies were calculated and these were Bonferroni corrected, due to multiple testing. Peptides with absolute frequencies 40, 45, 50 in the 10 μL LTP dataset showed no significant difference in the 10 μL HTP dataset. The data presented in Table 2 show the absolute frequencies (20, 25, 30, 35) with significant differences. The full dataset can be seen in table available at https://sid.erda.dk/share_redirect/eRcFQmhXEo (accessed on 27 January 2023). 

The table exemplifies the ability of HTP to make a distinction among peptides with the same frequency in the LTP datasets, as they were found to have different frequencies in the HTP sample. These results lead us to the conclusion that HTP sequencing can provide a relatively higher distinguishing capacity compared to LTP sequencing for the detection of peptide frequencies. 

### 2.5. The Peptide Composition of Datasets Shows Vast Dissimilarities for Different Sequencing Depths and Volumes

**Figure 3:**
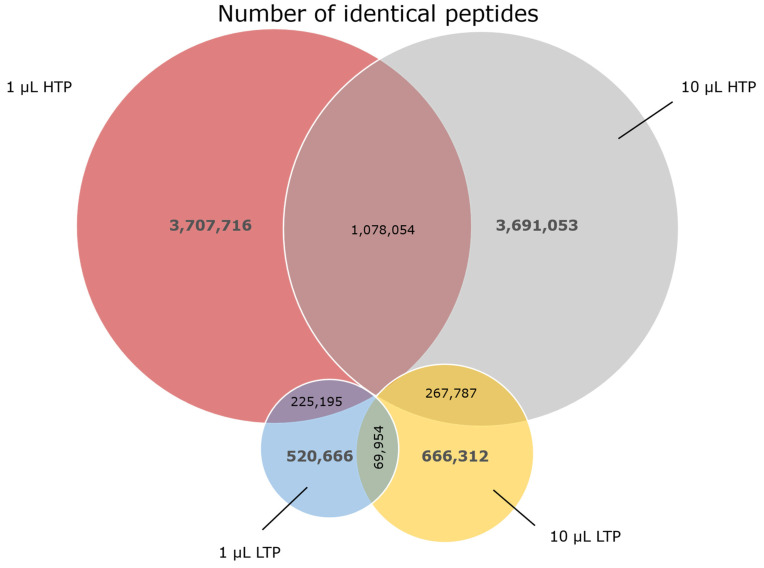
Illustration of the number of identical peptides between different datasets. A Venn diagram showing the number of identical peptides was made to compare samples with the same sequencing depth but different volumes and samples with the same volume but different sequencing depths. For each sample, the number of unique peptides is shown in grey, and the number of identical peptides is shown in black.

The sequence composition of the four datasets was compared by calculating the number of identical peptides (common between different datasets) in each sample. A Venn diagram showing the number of identical peptides for the different datasets can be seen in Figure 3. The two HTP datasets (1 and 10 μL) have 1.07 × 10^6^ identical peptides compared to 6.99 × 10^4^ which is the number of identical peptides in the two LTP datasets (1 and 10 μL). When comparing the 1 μL samples sequenced by HTP and LTP, 2.25 × 10^5^ peptides are identical and when comparing 10 µL samples sequenced by HTP and LTP 2.67 × 10^5^ peptides are identical. Overall, our data reveal that not only the peptide composition of the samples with different sequencing depths shows discrepancy (as expected), but also datasets with the same sequencing depth, but different input volumes, show a large discrepancy in their peptide composition. 

### 2.6. Different Sequencing Depths Show Discrepancies in the Position-Specific Frequency of Amino Acids 

**Figure 4:**
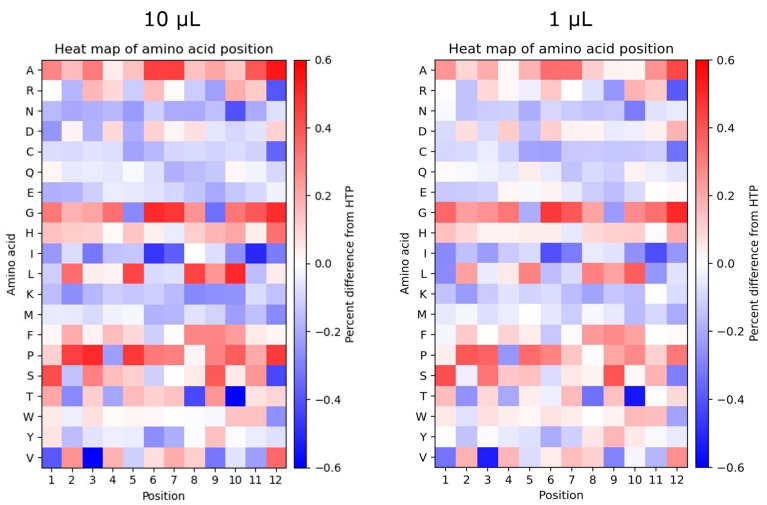
Percentage difference in the positional frequency of amino acids between samples with different sequencing depths. Heatmaps showing the percentage difference in the positional frequency of 10 µL LTP compared to 10 µL HTP (left) and 1 µL LTP compared to 1 µL HTP (right) for the 12 positions in the displayed peptide and for all amino acids. HTP datasets were used as references, accordingly, the scale indicates percentage difference from HTP. Overrepresentation of amino acids is shown in red, and underrepresentation of amino acids is shown in blue.

Differences in the position-specific frequency of amino acids were evaluated by comparing the 10 µL LTP dataset to the 10 µL HTP dataset (left heatmap) as well as 1 µL LTP to 1 µL HTP (right heatmap), as shown in Figure 4. Generally, it can be observed that differences exist across all positions and amino acid residues. The biggest differences can be observed for A, G, I, L, P, S, T, and V. The positional frequency of W appears to be equal for both LTP and HTP at positions 1–9 and shows minor over- and underrepresentation compared to HTP at positions 10–12. A, H, F, and P (except position 4) are generally overrepresented in LTP compared to HTP, whereas N, C, Q, E, I, K, and M are generally underrepresented compared to HTP. R, D, G, L, S, T, Y, and V indicate a combination of both over- and underrepresentation in different positions. 

When comparing the positional frequency for the samples with 1 µL to the samples with 10 µL, some differences can be observed, e.g., at positions 9 and 10 of A, position 4 of R, positions 1 and 11 of N, positions 3 and 5 of H, position 8 of I, position 11 of K, positions 2, 7 and 12 of S, positions 5, 6 and 11 of T, and multiple positions of D, E, L, W, and Y, among others. 

## 3. Discussion

Peptide phage display relies on the identification of genuine target binders in the recovered phages obtained from biopanning. Analysis of the exact composition of phage display peptide libraries plays a valuable role in the discovery of ligands with strong binding affinities to different biological targets [21]. To achieve this purpose, a sequencing platform capable of rigorous characterization of the composition and diversity of the peptide pool is crucial. High-throughput sequencing has been found to be suited for the analysis of the sequence composition and inherent diversity of phage display libraries [20,31]. In the current work, we made a side-by-side comparison of the capacity of two NGS platforms with different depths of sequencing in characterizing the composition and diversity of a phage display peptide library. Rodi et al. introduced two concepts to define the library diversity: Technical diversity (completeness) which corresponds to the percentage of possible members of the library existing at any absolute frequency and functional diversity which takes into consideration the absolute frequency of each distinct peptide in the library [24]. Consistent with this, Makowski and Soares proposed an analytical expression of the library diversity in which diversity is not only dependent on the number of peptides but also on the relative abundance of each peptide within the library [32]. Here, we used the number of unique sequences as a measure of library diversity. This definition is commonly used to refer to the observed diversity of phage display peptide libraries [16,19,25,26,27,28,29,30]. Our data indicated that sequencing depth has a vital impact on the ability to characterize the diversity of the library (Table 1 and Figure 1). A comparison of the two NGS modalities with LTP (720,000 absolute cleaned reads on average) and HTP (5,470,000 absolute cleaned reads on average) showed that the HTP platform can identify a significantly higher number of unique peptide sequences (unique cleaned reads in Table 1), thus revealing a much larger diversity of the library. It is worth mentioning that detecting the full diversity of phage display peptide libraries exceeds the capacity of current NGS platforms. The theoretical diversity of a 12-mer phage display peptide library can reach 4.1 × 10^15^. However, the maximum available diversity that can be experimentally achieved is 10^9^ [33]. This is mainly caused by limitations in the transformation efficiency of *E. coli* cells. The available diversity is calculated by titration of amplified phages after electroporation of the library into the host bacterium. Our previous report indicated that the diversity of the library (used in the current work) can be even lower due to the presence of stop codons, frameshifts, and clones without insert in the naïve library [20]. Consistent with this, the real diversity of phage display peptide libraries is expected to be within the range of 10^8^–10^9^. Given the restrictions of the current NGS platforms for determining the diversity of phage display peptide libraries, increased sequencing depth could provide a broader picture of the diversity and composition of the library. 

The LTP datasets indicate that the library contains a bigger percentage of the singleton population compared to HTP datasets (Figure 1). Also, LTP sequencing indicates a greater percentage of distinct sequences (according to the information in Table 1). A larger percentage of singletons, a smaller percentage of repeated peptides, and a larger percentage of distinct sequences characterize a higher quality of the library [28]. Our data provide evidence for the fact that these parameters are extremely dependent on the sequencing depth since LTP datasets falsely indicate a higher quality that is not reflected in the HTP datasets even though the library is the same. Our observation is in line with the previous findings on the Ph.D.^TM^-7 library where different sequencing depths were applied and the percentage of distinct sequences was different (58 % in one study [13] and 81 % in another study [19]. Some of these differences can also be attributed to the biases that occur in the different steps of sample preparation for NGS. Consequently, relying on data from LTP sequencing can be misleading for the evaluation of the library quality. This highlights the pitfalls associated with applying Sanger sequencing for investigation of the library quality and analyzing the diversity of a representative pool of peptides after biopanning. It is of interest to note that a larger percentage of singletons in the LTP datasets suggests a larger portion of the library to be dominated by peptides with the same frequency. This leads us to observe less heterogeneity in the library when using LTP sequencing. 

The investigation of the distribution of frequencies demonstrated that the HTP platform can detect a broader range of frequencies as well as a greater percentage of peptides with higher frequencies compared to the lower throughput platform (Figure 2). These results indicate that HTP sequencing can reveal a more heterogeneous distribution of peptide frequencies within the library and that LTP sequencing underestimates the library heterogeneity. This pattern is quite explicit for peptides with higher frequencies. Highly frequent peptides are important for the analysis of both unselected libraries and biopanning outputs. In the former, they might represent propagation-related target-unrelated peptides (Pr-TUPs) that are enriched during biopanning in a target-unrelated manner. In the latter, they might represent specific binders enriched during biopanning in a target-dependent manner. Hence, a sequencing platform with higher depth can improve the identification of both non-specific and specific peptides in phage display selections. Comparing peptides present in both HTP and LTP datasets showed that peptides having the same frequency in LTP sequencing in some cases resulted in different frequencies in HTP sequencing (Table 2). Hence, HTP has a relatively higher capacity for distinguishing peptide frequencies. The frequency of peptides is used as a basis to define the rank of sequences in NGS datasets and rank is a highly important selection criterion for peptides enriched during biopanning. This indicates that a higher depth of sequencing can provide a relatively more exact determination of peptide ranks in library selections.

The analysis of amino acid positional frequencies can be used in the evaluation of the library quality and diversity [24,34]. Furthermore, it offers valuable insights into the position-dependent enrichment of sequences during library selection that can be used to find sequence motifs in the peptide pool recovered from biopanning [16,35]. A comparison of the position-specific frequency plots obtained from the two different depths of sequencing revealed that the distribution of amino acids is not similar across the length of displayed peptides between different sequencing depths and shows some discrepancies between LTP and HTP datasets (Figure 4). Our data support the notion that using a sequencing platform with a higher throughput has the potential to be associated with less bias in the position-dependent sequence analysis of naïve libraries and position-dependent sequence enrichment of biopanning outputs, allowing for a more rigorous identification of enriched motifs. Also, calculating the number of identical peptides between different datasets indicated remarkable variation between LTP and HTP sequencing (as expected according to the significant difference in the diversity of these datasets) (Figure 3). Interestingly, different volumes of the same sequencing depth, although not displaying a notable difference in the diversity (Table 1), exhibit a substantial variation in their composition (Figure 3). The results highlight that major biases are associated with sampling from the naïve library, which can alter the peptide composition in the sampled pool and might cause dramatic changes in the pool of sequences arising from biopanning. The observed discrepancy in the composition of the sampled phage pool can be attributed to the huge diversity of the naïve unselected library. 

## 4. Materials and Methods

### 4.1. DNA Isolation from Phage Display Peptide Library

The Ph.D.^TM^-12 Phage Display Peptide Library (catalog #: E8111L) is based on the M13KE phage vector. It was purchased from New England Biolabs (Ipswich, MA, USA) (lot number: 10111202). This library expresses 12-mer peptides and a GGGS-linker on the N-terminal part of the minor coat protein pIII. The M13KE vector has been modified to allow a pentavalent display of peptides. The library is reported to have a complexity of 10^9^ independent clones. The single-stranded DNA (ssDNA) of the M13 phage was isolated from 1 µL and 10 µL of the Ph.D.^TM^-12 phage display peptide library using the NucleoSpin ^®^ Plasmid, Mini kit for plasmid DNA (Macherey-Nagel, Düren, Germany). The manufacturer’s protocol was used without the amplification of phage particles in *E. coli*.

### 4.2. Illumina Sequencing of the Phage Display Peptide Library 

To add sequencing adapters to DNA, PCR was performed with primers containing the adapter and the target-binding sequence (underlined) in a 50 mL reaction. The reaction volume consisted of Q5^®^ High-Fidelity 2X Master Mix (New England Biolabs, Ipswich, MA, USA), up to 100 ng of ssDNA, and 0.5 mM of forward and reverse primers. 

Forward primer: 5′-TCG TCG GCA GCG TCA GAT GTG TAT AAG AGA CAG ACC TCG AAA GCA AGC TGA TAA ACC G-3′.

Reverse primer: 5′-GTC TCG TGG GCT CGG AGA TGT GTA TAA GAG ACA GCT GTA GCA TTC CAC AGA CAG CCC-3′.

The PCR program used was conducted as follows: 98 °C for 30 s, 5 cycles of 98 °C for 10 s, 60 °C for 30 s, 72 °C for 20 s, and a final extension at 72 °C for 2 min. The PCR products were purified using GeneJET PCR Purification Kit (Thermo Fisher Scientific, Waltham, MA, USA) according to the manufacturer’s protocol. To allow for multiplexed sequencing, index PCR was performed using up to 25 ng of PCR product, 25 mL of Q5^®^ High-Fidelity 2X Master Mix, 5 mL of i5, and 5 mL of i7 Nextera XT indexing primers (Illumina, San Diego, CA, USA) in a 50 mL reaction volume. The cycling conditions were initial denaturation at 95 °C for 3 min, 8 cycles of 98 °C for 30 s, 60 °C for 30 s, 72 °C for 30 s, and 72 °C for 5 min as a final extension. The indexed PCR products were purified using the GeneJET PCR Purification Kit according to the manufacturer’s protocol. BioAnalyzer 2100 DNA 1000 Kit (Agilent, CA, USA) was used to verify the quality of the samples after each PCR reaction. 

Fluroskan^TM^ Microplate Fluorometer (Thermo Fisher Scientific, Waltham, MA, USA) was used with the Quant-iT^TM^ 1X dsDNA HS Assay Kit (Thermo Fisher Scientific, Waltham, MA, USA) to perform quality control of the samples. The indexed PCR products were pooled and sequenced using Illumina platforms. For lower throughput sequencing, the MiSeq Reagent Kit v2 and for higher throughput sequencing the NextSeq 500/550 Mid Output Kit v.2.5 (Illumina, San Diego, CA, USA) were used. For both platforms, 250-bp single-end sequencing was performed. The resulting individual base call (BCL) files were demultiplexed and the bcl2fastq software, provided by Illumina, was used to generate FASTQ files. The quality control, Illumina sequencing, and the generation of FASTQ files were conducted by the Center for Genomic Medicine, Rigshospitalet, Copenhagen University Hospitalet, Denmark. 

### 4.3. Analysis of Illumina Sequencing Data

To process the FASTQ files, a MATLAB script modified from [36] was used, as described previously in [20,37]. The script can be found at https://sid.erda.dk/share_redi-rect/ehmAUw0RrJ (accessed on 27 January 2023). The analysis was initiated by defining the borders of the region encoding displayed peptides in the Ph.D.^TM^-12 library and conversion of nucleotides into amino acids. Information about amino acid sequences, their occurrences, and their frequencies are found in the generated frequency-sorted matrix, in comma-separated values (.csv)-format. Reads containing ‘*’ or ‘X’ or reads that do not contain the GGGS-linker were removed. The raw data can be found at https://sid.erda.dk/cgi-sid/ls.py?share_id=HLpkNTfQhV (accessed on 27 January 2023). The resulting .csv files were used for subsequent analyses. The MATLAB script used to generate stacked bar plots can be found at https://sid.erda.dk/share_redirect/AX6yTHlTrX (accessed on 27 January 2023). Furthermore, an in-house Python script in Jupyter Notebook was used to calculate the positional frequency, one hot encoding was used for the amino acids, as described previously [20]. This is available at https://sid.erda.dk/share_redirect/D9nPiBpPE2 (accessed on 27 January 2023). Another Python script was used to evaluate the number of overlapping peptide sequences when comparing two samples, which is available at https://sid.erda.dk/share_redirect/dqxORN4rUG (accessed on 27 January 2023). 

## 5. Conclusions

Our findings suggest that the depth of the sequencing platform plays a pivotal role in the characterization of phage display peptide libraries. Our work provides insights into the limitations of lower throughput NGS. Compared to the traditional approach of Sanger sequencing, high-throughput sequencing strategies yield more valuable data on the compositional analysis of peptide pools. However, phage display researchers should be aware of the drawbacks of NGS platforms with lower depth, and the influence they might have on the analysis of phage display data. Lower sequencing depth can bear misleading information about the quality of unselected libraries and composition of biopanning outputs, which has substantial potential to misdirect further characterization of the in vitro selected peptides and final interpretations of phage display selection studies. We, therefore, recommend that phage display researchers thoroughly evaluate their experimental setup and draw particular attention to the constraints of the selected sequencing depth. 

## Figures and Tables

**Table 1:** Overview of the samples sequenced by different sequencing depths of NGS. The total unique reads refer to the number of reads with different peptide sequences and the total absolute reads refer to the total number of reads that each sample contains. The cleaned reads are the reads left after computational filtering of the sequencing data using a MATLAB script. The cleaned reads were used for all the analyses. HTP: higher-throughput, LTP: lower-throughput.

Sample	Unique Cleaned Reads	Unique Removed Reads (Number and Percentage)	Total Unique Reads	Absolute Cleaned Reads	Absolute Removed Reads (Number and Percentage)	Total Absolute Reads
HTP 1 μL	3.70 × 10^6^	7.03 × 10^5^ (15.95 %)	4.41 × 10^6^	5.52 × 10^6^	1.49 × 10^6^ (21.21 %)	7.01 × 10^6^
HTP 10 μL	3.69 × 10^6^	7.13 × 10^5^ (16.19 %)	4.40 × 10^6^	5.42 × 10^6^	1.52 × 10^6^ (21.93 %)	6.94 × 10^6^
LTP 1 μL	5.21 × 10^5^	7.89 × 10^4^ (13.17 %)	5.10 × 10^5^	6.23 × 10^5^	1.60 × 10^5^ (20.47 %)	7.84 × 10^5^
LTP 10 μL	6.66 × 10^5^	1.06 × 10^5^ (13.70 %)	7.72 × 10^5^	8.20 × 10^5^	2.15 × 10^5^ (20.80 %)	1.04 × 10^6^

**Table 2:** Comparison of the capacity to distinguish peptide frequencies between LTP and HTP platforms. Overview of peptide sequences with the same frequency in the 10 μL LTP dataset but varying frequencies in the 10 μL HTP dataset. Both the absolute and relative frequencies have been listed for each peptide sequence. Confidence intervals were calculated, and Bonferroni corrected.

Peptide Sequence	10 μL LTP			10 μL HTP		
	Absolute Frequency	Relative Frequency (%)	95% CI	Absolute Frequency	Relative Frequency (%)	95% CI
TLHFKPPNVTML	20	2.44 × 10^−3^	[5;35]	87	1.61 × 10^−3^	[56;118]
ERSMYWEDITPM				90	1.66 × 10^−3^	[58;122]
ASNGTDHTRTPF				189	3.49 × 10^−3^	[143;235]
AHTMQQISPNHC				85	1.57 × 10^−3^	[54;116]
SDSLFWNMMTDV				170	3.14 × 10^−3^	[127;213]
AWPPTGILPMLN				161	2.97 × 10^−3^	[119;203]
AMDRQPTWSVAN	25	3.05 × 10^−3^	[8;42]	144	2.66 × 10^−3^	[104;184]
HTSPRHYSSMSA				155	2.86 × 10^−3^	[114;196]
NTVYAQPTGVLS				120	2.21 × 10^−3^	[84;156]
TAPRPQSILNGL				128	2.36 × 10^−3^	[90;166]
LLPTGNVLENFP				120	2.21 × 10^−3^	[84;156]
TPPQISTPTQPV	30	3.66 × 10^−3^	[12;48]	133	2.45 × 10^−3^	[95;171]
NLNDSYGLSSDR				256	4.72 × 10^−3^	[203;309]
NLVSTGLAYQSL	35	4.27 × 10^−3^	[15;55]	163	3.01 × 10^−3^	[121;205]
GNMGYMRPGHNN				160	2.95 × 10^−3^	[118;202]

## Data Availability

The data presented in this study is openly available at https://sid.erda.dk/cgi-sid/ls.py?share_id=DVXJPIq0QL (accessed on 27 January 2023).
